# Characterization of a novel large deletion and single point mutations in the *BRCA1 *gene in a Greek cohort of families with suspected hereditary breast cancer

**DOI:** 10.1186/1471-2407-4-61

**Published:** 2004-09-07

**Authors:** Ioulia Belogianni, Angela Apessos, Markos Mihalatos, Evangelia Razi, Stefanos Labropoulos, Andreas Petounis, Vasiliki Gaki, Antonios Keramopoulos, Nikos Pandis, Kyriacos Kyriacou, Andreas Hadjisavvas, Paris Kosmidis, Drakoulis Yannoukakos, Georgios Nasioulas

**Affiliations:** 1 Molecular Biology Research Center HYGEIA «Antonis Papayiannis», DTCA HYGEIA, 15123 Maroussi, Athens, Greece; 2 1^st ^Pathology – Oncology Clinic, DTCA HYGEIA, 15123 Maroussi, Athens, Greece; 3 Breast Cancer Unit, Iaso Women's Hospital, 15123 Maroussi, Greece; 4 Department of Genetics, "Saint Savas" Anticancer Hospital, 11522 Athens, Greece; 5 Department of Electron Microscopy/Molecular Pathology, The Cyprus Institute of Neurology and Genetics, 1683 Nicosia, Cyprus; 6 2^nd ^Pathology – Oncology Clinic, DTCA HYGEIA, 15123 Maroussi, Athens, Greece; 7 Molecular Diagnostics Lab, I/R-RP, National Center for Scientific Research "Demokritos" 15310 Athens, Greece

## Abstract

**Background:**

Germline mutations in *BRCA1 *and *BRCA2 *predispose to breast and ovarian cancer. A multitude of mutations have been described and are found to be scattered throughout these two large genes. We describe analysis of *BRCA1 *in 25 individuals from 18 families from a Greek cohort.

**Methods:**

The approach used is based on dHPLC mutation screening of the *BRCA1 *gene, followed by sequencing of fragments suspected to carry a mutation including intron – exon boundaries. In patients with a strong family history but for whom no mutations were detected, analysis was extended to exons 10 and 11 of the *BRCA2 *gene, followed by MLPA analysis for screening for large genomic rearrangements.

**Results:**

A pathogenic mutation in *BRCA1 *was identified in 5/18 (27.7 %) families, where four distinct mutations have been observed. Single base putative pathogenic mutations were identified by dHPLC and confirmed by sequence analysis in 4 families: 5382insC (in two families), G1738R, and 5586G > A (in one family each). In addition, 18 unclassified variants and silent polymorphisms were detected including a novel silent polymorphism in exon 11 of the *BRCA1 *gene. Finally, MLPA revealed deletion of exon 20 of the *BRCA1 *gene in one family, a deletion that encompasses 3.2 kb of the gene starting 21 bases into exon 20 and extending 3.2 kb into intron 20 and leads to skipping of the entire exon 20. The 3' breakpoint lies within an *Alu*Sp repeat but there are no recognizable repeat motifs at the 5' breakpoint implicating a mechanism different to *Alu*-mediated recombination, responsible for the majority of rearrangements in the *BRCA1 *gene.

**Conclusions:**

We conclude that a combination of techniques capable of detecting both single base mutations and small insertions / deletions and large genomic rearrangements is necessary in order to accurately analyze the *BRCA1 *gene in patients at high risk of carrying a germline mutation as determined by their family history. Furthermore, our results suggest that in those families with strong evidence of linkage to the *BRCA1 *locus in whom no point mutation has been identified re-examination should be carried out searching specifically for genomic rearrangements.

## Background

Germ line mutations in the *BRCA1 *and *BRCA2 *genes predispose individuals to breast and ovarian cancer. The lifetime risk of breast cancer in female carriers of a *BRCA1 *mutation is 60–80% while that of ovarian cancer is 20–40%. The median age of diagnosis of breast cancer is 42 years, i.e. 20 years earlier than the median of unselected women in the U.S.A. and Western Europe [1]. *BRCA1 *is a large gene with 22 coding exons encoding a 220 kD protein [2] that functions in maintaining genomic integrity and in transcriptional regulation [3,4].

A multitude of mutations scattered throughout the 5592 bp coding sequence have been described. In particular, germ line mutations in *BRCA1 *have been identified in 15–20% of women with a family history of breast cancer and 60–80% of women with a family history of breast and ovarian cancer [5,6]. The percentage of mutations identified is strongly dependent on the population studied, with strong founder effects evident in some populations [7-9]. The vast majority of mutations described to date are point mutations and small insertions and deletions . Such mutations are detected by PCR-based screening methods such as the Protein Truncation Test (PTT), Single Strand Conformational Polymorphism (SSCP), Denaturing Gradient Gel Electrophoresis (DGGE), heteroduplex analysis (HA) and more recently denaturing high performance liquid chromatography (dHPLC) with varying degrees of sensitivity for each method. Direct DNA sequencing is used in order to confirm and characterize mutations detected by any of these approaches [10,11]. While in the early studies for mutation detection only single point or small insertion/deletion mutations were screened for, recent studies have shown that genomic rearrangements are also a common type of mutation in the two genes accounting for 10–30 % of all mutations identified in some populations [12-16].

In this study we document and extend previous work suggesting the necessity of screening for large genomic rearrangements in a complete program for mutation detection of the *BRCA1 *gene by characterizing a deletion encompassing 3.2 kb of the gene including exon 20. In addition, we add more evidence supporting pathogenicity of a previously described variant, G1738R, which seems to be specific to the Greek population [17].

## Methods

### Patients

*BRCA1 *patients and their families were referred through the Oncology Departments of Hygeia Hospital and other hospitals throughout Greece. Patients were included on the basis of affected family members, types of cancer present in the family and the age at diagnosis of breast cancer in the proband. The families were subdivided into high risk when multiple cases of breast and ovarian cancer were diagnosed, medium risk if there were only 2–3 cases of breast cancer, and low risk in isolated cases of breast cancer with diagnosis before the age of 40 years. The study population consisted therefore of 12 high risk families, 2 with medium risk, and 4 families at low risk as determined by their cancer history. Ethical approval was obtained from the hospital's advisory committee and all patients signed informed consent. Screening has been completed in 25 individuals from 18 families. Testing was initially carried out on DNA from an affected family member and upon detection of an inactivating mutation the rest of the family members were directly tested for this mutation.

### DNA and RNA isolation

Genomic DNA and RNA were purified from peripheral blood leukocytes using standard extraction protocols.

### PCR amplification

The complete coding sequence of *BRCA1 *including splice junctions was amplified by PCR. Similarly, exons 10 and 11 of the *BRCA2 *gene were amplified in 3 of the patients. Primers used have been chosen from the BIC database . In addition primers: mBRCAF: GAG TTT GTG TGT GAA CGG ACA CTG and mBRCAR: GTG CCA AGG GTG AAT GAT GAA AGC, designed during the course of this study, were used for amplification of cDNA from the patients found to carry a deletion of exon 20 of the *BRCA1 *gene.

Reactions of 50 μl were heated on a PTC-200 MJ Research Thermocycler (MJ Research Inc., USA) at 95°C for 5 min then cycled 35 times of denaturation at 95°C for 40 sec, annealing at the appropriate temperature for 30 sec and extension at 72°C for 30 sec, followed by a final extension step at 72°C for 6 min. Reaction mixture was 20 mM TrisHCl (pH 8.4), 50 mM KCl, 1.5 mM MgCl_2_, 200 μM each dNTP, 1.5 U Taq DNA polymerase (Invitrogen, Netherlands) or 2.5 U Optimase polymerase (Transgenomic, Inc., USA) and 12.5 pmol of each primer.

### dHPLC analysis

The WAVE DNA Fragment Analysis System (Transgenomic, Inc., USA) and associated WAVE-Maker™ software were used as previously described [18].

### Sequence analysis

Purification of the PCR products was performed using the Concert Rapid PCR purification or gel extraction system kits (Marligen Biosciences INC, U.S.A.). Automated cycle sequencing for both strands was performed with the ABI Prism^® ^310 Genetic Analyzer using the Big-Dye Terminator Cycle Sequencing Kit. Sequences obtained were aligned, using Sequencher^® ^PC software, with normal sequences from Genbank (*BRCA1*: L78833, *BRCA2: *U43746) and examined for the presence of mutations. All nucleotide numbers refer to the wild-type cDNA sequence of *BRCA1 *as reported in GenBank (accession number U14680).

### Multiplex Ligation – dependent PCR Amplification (MLPA)

MLPA was carried out using the P002_BRCA1 kit (MRC-Holland, Netherlands) as instructed by the manufacturer. Fragment analysis was carried out on ABI Prism^® ^310 Genetic Analyzer (Applied Biosystems, USA) using TAMRA-500 (Applied Biosystems, USA) as size standard. A peak pattern of 34 peaks ranging in size from 127 to 454 nt is obtained [19].

### Long PCR

The deletion in *BRCA1 *exon 20 was confirmed by long PCR using the GeneAmp XL PCR System (Applied Biosystems, U.S.A.) according to the manufacturer's instructions. PCR was carried out in 50 μl reactions consisting of 1 × buffer II (supplied with the enzyme), 12 pmole each primer, 0.2 mM each dNTP and 2.4 U *rTth *DNA polymerase, XL (Applied Biosystems, U.S.A.). After 2.5 min denaturation at 95°C PCR was carried out for 19 cycles of 95°C for 30 sec, 58°C for 30 sec and 68°C for 8 min followed by 15 cycles of 95°C for 30 sec, 58°C for 30 sec and 68°C for 4 min with a time increment of 10 sec per cycle. A final extension step was carried out at 72°C for 10 min. PCR products were separated by agarose gel electrophoresis and visualized by EtBr staining.

### RT-PCR

Total RNA was extracted from whole blood of patients from family D using Trizol (Life Technologies, USA) according to the manufacturer's instructions. First strand synthesis was performed by denaturing approximately 500 – 1000 ng total RNA, random hexamers (5 μM final concentration) for 4 min at 70°C, followed by snap freezing on ice and addition of dNTPs (0.5 mM final concentration), 1 U/μl recombinant RNase inhibitor (Invitrogen, Netherlands) and 200 U MMLV reverse transcriptase (Invitrogen, Netherlands). The mixture was incubated at 37°C for 1 hour followed by denaturation of the enzymes at 95°C for 5 min. 4 μl of cDNA were used for subsequent PCR amplification.

## Results

A total of 18 families, 12 of which were at high risk of having hereditary breast cancer, have been examined for mutations at the *BRCA1 *locus. A pathogenic mutation was identified in five families, where four distinct mutations have been observed. In addition, 18 polymorphisms, including a novel silent polymorphism in exon 11 of the *BRCA1 *gene, have been detected (Table 1). Furthermore, in 3 of the families exons 10 and 11 of *BRCA2 *were analyzed. Table 1 summarizes the results of single nucleotide variants detected in this study. All of the variants have been identified by dHPLC and characterized by sequencing.

In family A there were four cases of breast cancer affecting four successive generations. The proband was diagnosed with breast cancer at the age of 45. The most frequently occurring mutation, 5382insC in exon 20, was identified. Analysis of the proband's daughter in whom cancer developed at the age of 38 revealed that she also carried the mutation. The same mutation was also identified in family E where breast cancer was diagnosed in 3 members in three successive generations at 42, 37 and 41 years (data not shown).

In family B there were seven cases of breast cancer and one case of colorectal cancer at age 50. Mutation analysis at the *BRCA1 *locus revealed a missense mutation 5331 G > A. The mutation results in substitution of a Glycine by an Arginine at codon 1738. The mutation was not detected in the patient's unaffected sister. Unfortunately, no other family members were available for analysis.

In family C there were 4 cases of breast cancer in addition to cancer of the larynx, ovaries, lung and the genitals. A single base substitution, G > A at nucleotide 5586 was identified. This mutation causes a splicing defect resulting in a protein lacking exon 23. Unfortunately, no DNA was available from other family members in order to test the correlation of this mutation with the other tumor types.

**Figure 1:**
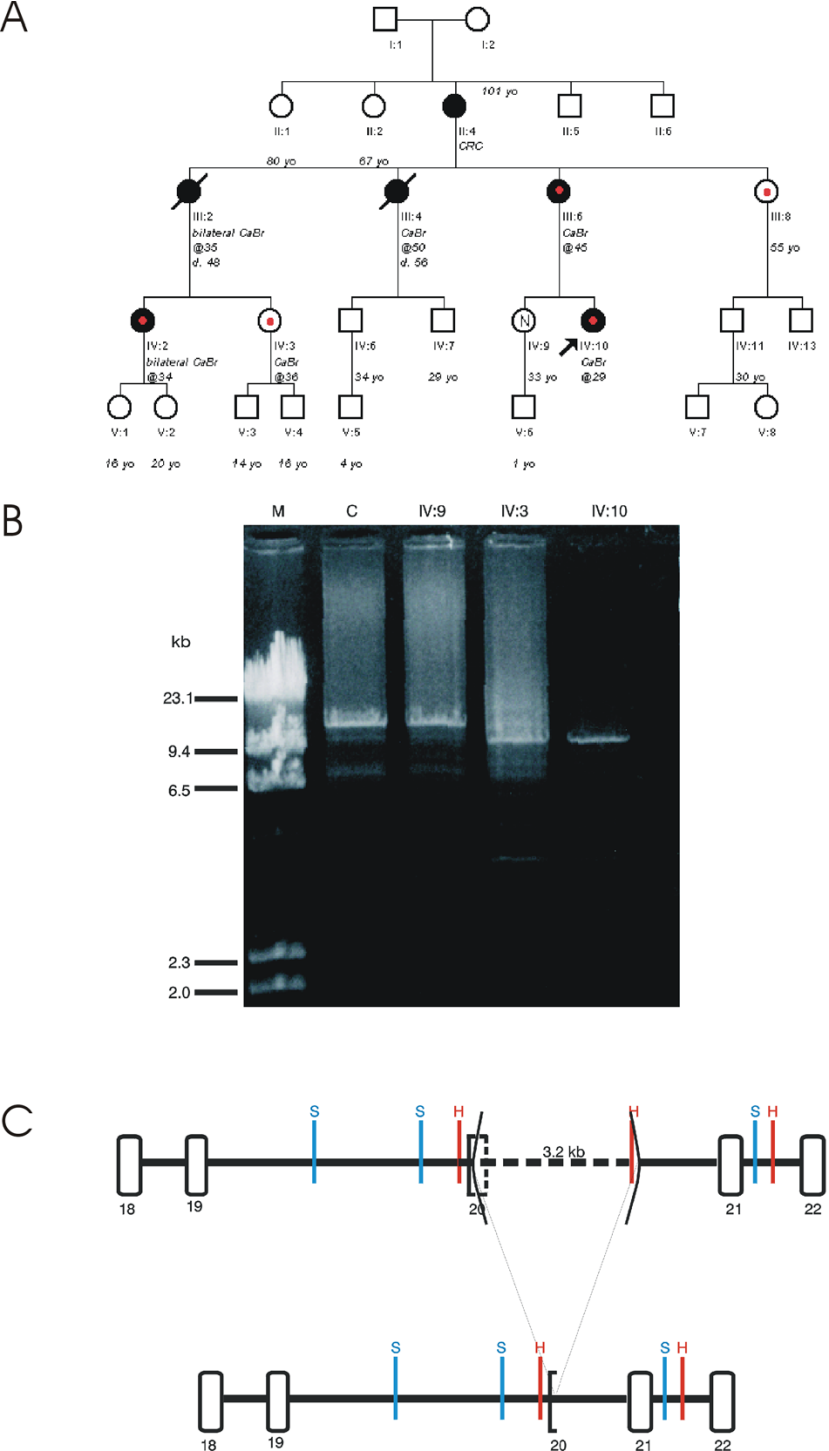
Family D in whom deletion of exon 20 of the *BRCA1 *gene was detected. a. Pedigree of family D. Black symbols indicate individuals with cancer. The proband is indicated by an arrow. A red circle inside a symbol indicates individuals found to carry the mutation. (CaBr=breast cancer, CRC=colorectal cancer d.=age of death, N=individual tested for the family mutation and was found to be of normal genotype). b. Long PCR of DNA from three individuals in family D. (M=molecular size marker (λ/HindIII, New England Biolabs, U.S.A.), C=normal control, numbers denote the identifier in the pedigree). c. Schematic representation (not to scale) of the fragment of *BRCA1 *gene amplified by Long PCR showing the position of exons, restriction endonuclease cleavage sites and the deletion. Empty boxes indicate exons. Vertical colored bars indicate restriction sites: blue (S) = *Sma*I. Red (H) = *Hind*III.

In Family D (Figure 1a) there were 5 cases of breast cancer with age at onset ranging from 29 to 50 years. In addition there was one individual who had CRC. Sequencing of the complete coding region of *BRCA1, *failed to reveal a mutation. This prompted the analysis of other genes, namely *BRCA2 *and *p53 *in order to try and characterize the phenotype in this family, but no mutation was identified. For this reason, we decided to use the MLPA technique [19] to screen the proband for genomic rearrangements which have been shown to be responsible for a large proportion of *BRCA1 *mutations. MLPA analysis of the proband (IV:10 in figure 1a) revealed deletion of exon 20 of the *BRCA1 *gene. This was confirmed by Long PCR (Figure 1b) and RT – PCR. Sequencing of PCR products generated by RT-PCR confirmed absence of the entire exon 20 in the mRNA of the patient. Subsequently, a number of restriction endonucleases were employed in order to narrow down the deletion breakpoints and facilitate their characterization. A *Sma*I-generated irregular fragment was finally isolated from agarose gel and sequenced revealing a deletion of 3.2 kb starting 21 bases into exon 20 and extending 3.2 kb into intron 20 (Figure 1c). The 3' breakpoint lies within an *Alu*Sp repeat but there are no recognizable repeat motifs at the 5' breakpoint. The deletion was also found in 4 relatives of the proband (Figure 1a) who were tested, two of whom had breast cancer. The other two relatives had not yet developed cancer at the ages of 36 and 55 at the time of testing and are therefore assumed to be pre – symptomatic carriers. Finally, the proband's sister, who at the time of testing had not developed cancer at the age of 33 was found not to carry the mutation.

## Discussion

A total of 18 breast cancer families have been examined for mutations at the *BRCA1 *locus. In three of the families analysis was extended to exons 10 and 11 of the *BRCA2 *gene. A pathogenic mutation in *BRCA1 *was identified in 5/18 (27.7 %) families, where four distinct mutations have been observed. In addition, 18 polymorphisms have been found to be present in more than one family.

In family B a missense mutation, 5331 G > A, was identified. The exact effect of this single amino acid change, G1738R, on the protein function is unclear. The altered glycine is located on the surface of the coil structure of the BRCT linker region and the mutation therefore may disrupt the interface or affect protein interaction [20]. This mutation has been previously described in 4 unrelated Greek patients [21]. An alternative mutation affecting the same amino acid, G1738E, has been shown to result in loss of function of the protein *in vitro *[22,23]. Based on this information, the family history of the individual described here and absence of the mutation in the proband's unaffected sister we hypothesize that the mutation is pathogenic although additional data are needed.

Our results indicate, as has been documented by others that family history is the major determinant of the risk of breast cancer. As can be seen in Table 1 in all families where a pathogenic mutation was identified there were at least 3 cases of breast cancer. In addition, the extremely high risk suggested by the family history in some cases prompted a more intense analysis of further genes and approaches in an attempt to characterize the underling reason for such a family history. This was the case in family D where sequencing of the complete coding region of *BRCA1, *failed to reveal a mutation. This prompted the analysis of other genes, namely *BRCA2 *and *p53*, but without finding a mutation. For this reason, we decided to use the MLPA technique [19] to screen the proband for genomic rearrangements which have been shown to be responsible for a large proportion of *BRCA1 *mutations [12-16]. This led to the identification of deletion of an entire exon of the *BRCA1 *gene, namely exon 20.

Deletion of exon 20 has been previously described in an Italian family [12]. However, the authors of that report have localized the breakpoints of the deletion in the two flanking introns [12]. In the deletion described here the 5' breakpoint is localized inside exon 20 (21 bp upstream of the splice donor site) while the 3' breakpoint is located 3.2 kb into intron 20 within an *Alu*Sp repeat. The deletion described here, therefore, is different to the majority of rearrangements described so far for the *BRCA1 *gene, since they have been shown to result from homologous recombination of *Alu *repeats [24,25]. In this case, the 5' breakpoint does not correlate with any recognizable repeat motifs suggesting a repeat – independent recombination mechanism at play.

In 2/5 pedigrees in whom a pathogenic mutation was identified there was one case each of colorectal cancer (CRC). In particular, in family D (Figure 1a) the disease seems to have originated from a CRC patient. Data on the correlation between *BRCA1 *mutations and the risk of CRC are not conclusive. Two recent studies carried out on Ashkenazi Jewish patients suggest that there is no correlation [26,27]. Other reports carried out on more diverse populations suggest 2- to 3-fold elevated risks of CRC among first-degree relatives of *BRCA1 *mutation carriers [28,29]. Unfortunately, there was no DNA available for analysis from the two CRC patients in the two families.

A further interesting point observed in this study is the identification of two *BRCA1 *unaffected mutation carriers at the ages of 39 and 55. The penetrance and age of onset of disease in *BRCA1 *mutation carriers is variable. Various reports have suggested the existence of modifying genetic and environmental factors on the penetrance of *BRCA1 *and *BRCA2 *mutation carriers [30,31]. In this respect it is interesting to examine this large pedigree for such modifying factors.

## Conclusions

To our knowledge this is the first report of a genomic rearrangement identified as the underlying mutation of *BRCA1 *in a Greek family. Although our sample group is quite small identification of 1 genomic rearrangement in 5 mutations detected, suggests that in yet another population this type of mutations contribute to the *BRCA1 *mutation spectrum. This therefore warrants use of a combination of techniques capable of identifying both single base mutations in addition to large genomic rearrangements. In this respect, we have found that use of dHPLC for single base mutations and MLPA for large genomic rearrangements is a reliable combination for use as an initial screening step followed by sequencing for characterization of the mutations identified.

Furthermore, our results suggest that in those families with strong evidence of linkage to the *BRCA1 *locus in whom no point mutation has been identified re-examination should be carried out searching specifically for genomic rearrangements.

## List of abbreviations

**dHPLC **denaturing High Performance Liquid Chromatography

**MLPA **Multiplex Ligation – dependent PCR Amplification

**PCR **Polymerase Chain Reaction

**RT-PCR **Reverse Transcription Polymerase Chain Reaction

**CRC **Colorectal Cancer

## Competing interests

None declared.

## Authors contributions

IB carried out mutation detection by dHPLC and sequencing for the patients analyzed. AA carried out MLPA, the molecular characterization of the deletion, and drafted the manuscript. MM contributed in the molecular studies and preparation of the manuscript and figures, ER, SL, AP, VG, AK NP, PK and DY provided the patient material, diagnosis and management,, KK and AH carried out molecular genetic analysis of the p53 gene, GN conceived of the study, and participated in its design and coordination. All authors read and approved the final manuscript.

## Pre-publication history

The pre-publication history for this paper can be accessed here:



## Figures and Tables

**Table 1:** Variants identified in the *BRCA1 *and *BRCA2 *genes during this study.

**Mutation**						
**Family No**	**Total No of CaBr^1^**	**No of CaOv^2 ^cases**	**Exon**	**Nucleotide**	**Effect**	**Other cancers (age at onset)**

**A**	4	-	20	5382ins C	Fs^3^	-
**B**	7	-	20	5331 G > A	G1738R	Colorectal (50)
**C**	4	1	23	5586G > A	Splicing	Larynx Lungs Genitalia
**D**	5	-	20	Deletion of the entire exon	Protein missing 20 a.a. of the BRCT-linker domain	Colorectal
**E**	3	-	20	5382ins C	Fs^3^	-

**Benign polymorphisms**						

	**Exon**	**Codon**	**Nucleotide Change**		**Consequences**	**No of families**

***BRCA1***	**IVS 4**	IVS4-19	C > A		-	1
	**IVS 7**	IVS7-34	C > T		-	2
	**11**	693	2196G > A		Asp > Asn	2
		694	2201C > T		Ser > Ser	5
		771	2430T > C		Leu > Leu	6
		871	2731C > T		Pro > Leu	3
		1038	3232A > G		Glu > Gly	7
		1040	3238G > A		Ser > Asn	1
		**1145^4^**	**3544T > C**		**Val > Val**	**1**
		1150	3567C > T		Pro > Ser	1
		1183	3667A > G		Lys > Arg	7
	**13**	1436	4427T > C		Ser > Ser	4
	**16**	1613	4956A > G		Ser > Gly	1
	**24**	1829	5606G > A		Glu > Glu	2
***BRCA2***	**10**	372	1342A > C		Asn > His	1
	**11**	1132	3624A > G		Lys > Lys	2
		1269	4053T > C		Val > Val	1
	**14**	2414	7470A > G		Ser > Ser	1

^1 ^CaBr = Breast Cancer

^2 ^CaOv = Ovarian Cancer

^3 ^fs = frame-shift mutation

^4 ^novel polymorphism identified in this study
